# Description of *Perithreticusneglectus* sp. n. from the West Usambara Mountains, Tanzania (Diptera, Psychodidae)

**DOI:** 10.3897/BDJ.10.e81205

**Published:** 2022-05-05

**Authors:** Gunnar Mikalsen Kvifte

**Affiliations:** 1 Nord University, Steinkjer, Norway Nord University Steinkjer Norway

**Keywords:** Psychodidae, Diptera, moth flies, Tanzania, West Usambara Mountains

## Abstract

**Background:**

The Psychodinae of the Afrotropical Region remain poorly understood. Slightly under 200 species have been described, but many countries have received very little attention from collectors and even countries where significant collection efforts have taken place have rarely had their collections studied in detail by specialist taxonomists.

**New information:**

*Perithreticusneglectus* sp. n. is described from the West Usambara Mountains, Tanzania, based on a male specimen collected in 1990. The new species is similar to *Perithreticusanderseni* Kvifte, 2015, which occurs in the same forest reserve, but can be separated by several genitalic characters, including the hypandrium well-developed with sclerotised anterior and posterior margins, gonocoxites narrower, the gonostyles with the slender apex shorter, the parameres shorter without pronounced basolateral projections and the surstylus with slightly fewer tenacula. The world fauna of *Perithreticus* now comprises five described species, of which two occur in the Afrotropical Region.

## Introduction

The Psychodidae material, collected by the University of Bergen's Tanzania expeditions in the early 1990s (described in Andersen and Johanson 1992 and Wagner and Andersen 2007), has been partially treated by Wagner and Andersen (2007), Kvifte (2014), Kvifte (2015) and Kvifte and Andersen (2019) and a total of 17 species have been described from there. This is likely not reflecting the true diversity of the West Usambara Mountains, or even the diversity present in the collected material and the collection thus is continuously re-examined.

The genus *Perithreticus* Vaillant, 1973 was described for two Nearctic species characterised by surstylus with tenacula in an apical row, named and briefly characterised by Vaillant (1973) in his revision of Nearctic Trichopsychodina (as "Telmatoscopini of the Threticus group"). The genus was later revised and redefined by Kvifte (2015), who removed *Psychodajonesi* Quate, 1955 from the genus, described a new Afrotropical species and speculated that *Philosepedonforcipata* Quate & Quate, 1967 and *Philosepedonpectinata* Quate & Quate, 1967 from Indonesia (Papua) might also belong to *Perithreticus*. Finally, Kvifte et al. (2016) described two *Perithreticus* species from Cuba and Costa Rica and presented the most recent diagnosis of the genus.

In the present paper, I describe a second species of *Perithreticus* from the West Usambara Mountains, which is also the second species of *Perithreticus* to be described from the Afrotropical Region.

## Materials and methods

The specimen was dissected, macerated in potassium hydroxide (KOH) and mounted in Canada balsam on a slide. Illustrations and measurements were made using a Leitz Diaplan 20 compound microscope with a drawing tube and an ocular micrometer. Measurements are given in μm with an accuracy of 2.5 μm, except wings which are given in mm with an accuracy of 25 μm. Morphological terminology follows Kvifte and Wagner (2017). The specimen is housed in the entomological collections of the University Museum of Bergen (ZMUB).

## Taxon treatments

### Perithreticus neglectus

Kvifte, 2022 sp. n.

E57E4E34-C603-54C0-A95C-376F23B4106E

0C6FEF11-AC20-413F-9167-A16A38322558

#### Materials

- **Type status:** Holotype. **Occurrence:** catalogNumber: B-10800.; **Taxon:** scientificName: Perithreticusneglectus Kvifte, 2022; order: Diptera; family: Psychodidae; genus: Perithreticus; specificEpithet: neglectus; **Location:** continent: Africa; country: Tanzania; stateProvince: Tanga; locality: Mazumbai forest reserve; verbatimLocality: Tanzania: Tanga Region, W. Usambara Mts., Mazumbai, Loc. B; decimalLatitude: -4.800; decimalLongitude: 38.500; geodeticDatum: WGS8; **Identification:** identifiedBy: Kvifte, Gunnar Mikalsen; **Event:** samplingProtocol: Malaise trap; year: 1990; month: 11; day: 3; verbatimEventDate: 03/11/1990; fieldNumber: B; **Record Level:** institutionCode: ZMUB

#### Description

**Figure 1.**
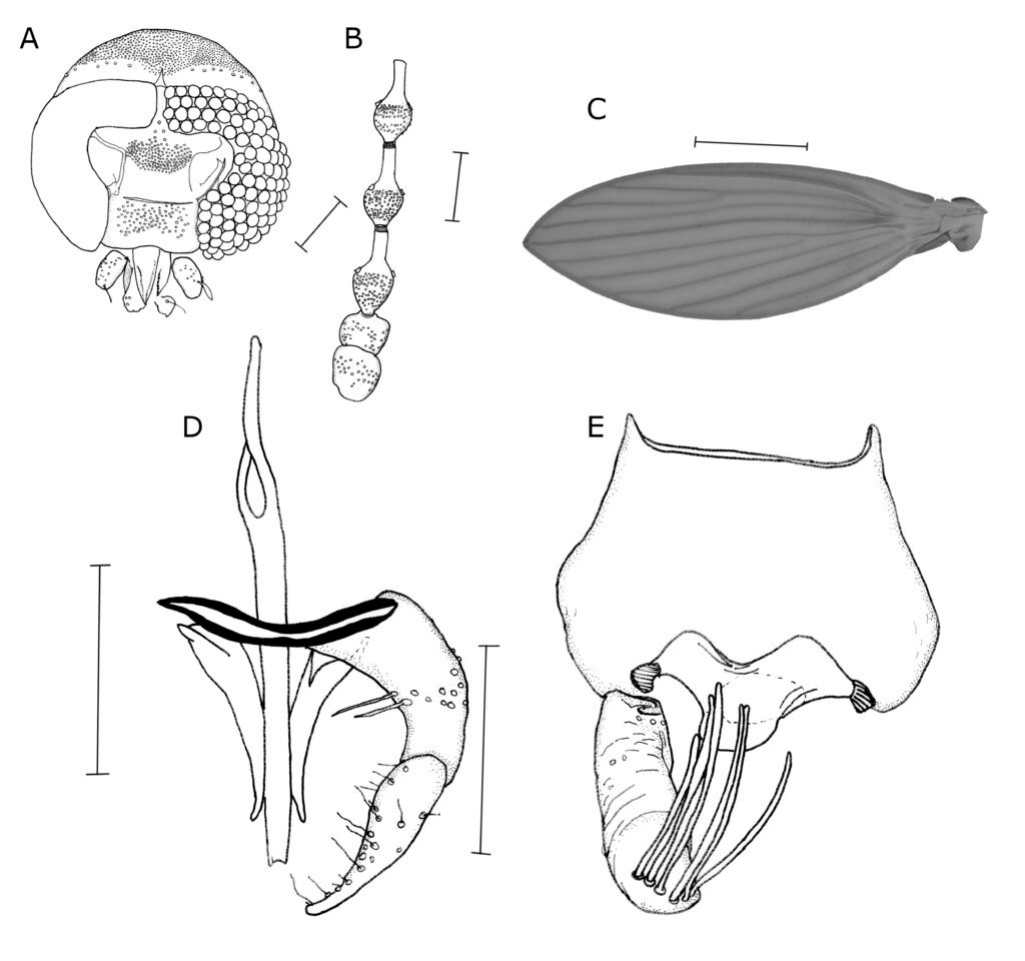
Morphological illustrations of *Perithreticusneglectus* sp. n. **A** head; **B** antenna; **C** wing; **D** gonopods, aedeagus and parameres; **E** epandrium and proctiger. Scale bars: 100 µm (A, B, D, E), 500 µm (C).

**Adult male (n=1). Head** (Fig. 1A) longer than wide; vertex about a fifth of total head length; eye bridge of four facet rows, separated by 0.5 facet diameters; with single row of 8-9 postocular setae; interocular area slightly broader anteriorly; interocular suture triangular; frontal patch of setae alveoli crown-shaped with median posterior extension reaching anteriormost row of eye bridge; length of first palp segment 67.5, other palpomeres not preserved; labellum bulbous and setose; only single antenna with three flagellomeres preserved in specimen (Fig. 1B), scape stoutly barrell-shaped, of equal width; pedicel stout spheroid, wider than long; flagellomeres 1-3 symmetrical nodiform with paired ascoid insertions, ascoids lost; length of scape, pedicel and first three flagellomeres 70, 62.5, 130, 125, 125; **Thorax** with anepisternum with trapezoid hair patch, anterior spiracle with prolonged U-shaped posterior suture delimiting it from anepisternal hair patch; anepimeron triangular with sinusoid lower margin, ventral suture of anepimeron reaching about halfway into sclerite; posterior spiracle with operculum evenly setose; mid-coxa with anteromesal field of setae; **Wing** (Fig. 1C) elipsoid, 2.25 mm long, 0.75 mm wide; membrane only with micropilosity; area between C and R_1_ infuscate; hyaline field below R1 reaching level of medial fork; radial fork clearly distad of medial fork and around same level as CuA; outlines of R_5_ and M_4_ more strongly sclerotised than other veins; origin of R_5_ with dark spot; jugum broadly angular U-shaped.

**Terminalia** (Fig. 1D, E) symmetrical, hypandrium with only anterior and posterior margin sclerotised, glabrous or membranous medially; gonocoxites reniform with parabasal process broadly triangular, meeting medially; single band of setae present, covering medial 5^th^, gonocoxal condyles with triangular plate laterally and narrow strip-like plate medially; gonostyli bluntly acuminate, covered in spiniform sensilla, with subapical trichiform sensilla nearly the length of broad gonostylar base; aedeagus with basiphallus short, about a third of length of distiphallus, divided into two phallomeres that fuse after a drop-shaped basal aperture, forming long parallel-sided rod-like distiphallus; parameres elongate curved subtriangular with lateral margins concave and mesal margins convex, blunt apically, basally with small pointed lateral processes; aedeagus reaching further than parameres; epandrium (Fig. 1E) wider than long; anteroventral surface covered in hairs; subepandrial sclerite only discernible as submedian keel in specimen; surstylus cylindrical, apparently curved, apically with 6-7 tenacula in single transverse row; hypoproct mostly naked, but with distal margin finely pilose, M-shaped; epiproct subrectangular with rounded corners; proctiger laterally with small sclerites (vestigial cerci?) pointing towards surstyli.

#### Diagnosis

Can be recognised by the following combination of characters: radial fork distad of medial fork, hypandrium with large unsclerotised area medially, aedeagus parallel-sided with triangular parameres shorter than aedeagus, parameres with triangular basolateral expansions poorly developed, surstylus with 6 tenacula, gonostyle with subapical trichiform sensilla (see also key in Kvifte et al. 2016).

#### Etymology

From Latin neglectus, "overlooked", "neglected", referring to the specimen not being included in the initial revision of Afrotropical Psychodini by Kvifte (2015).

#### Distribution

Only known from the type locality in the Mazumbai Forest Reserve, West Usambara Mountains, Tanzania.

## Supplementary Material

XML Treatment for Perithreticus neglectus
